# Supplementary material for: Recent Increases in Influenza-Related Hospitalizations, Critical Care Resource Use, and In-Hospital Mortality: A 10-Year Population-Based Study in South Korea
Source: J Clin Med. 2022 Aug 21;11(16):4911. doi: 10.3390/jcm11164911 (PMC9410240; doi:10.3390/jcm11164911)
Supplement: Supplementary file 1 [file jcm-11-04911-s001.zip › jcm-1873802-supplementary.pdf]

Supplementary Materials

Recent Increases in Influenza-Related Hospitalizations, Critical Care Resource Use, and In-Hospital Mortality; a 10-Year Population-Based Study in South Korea

Table S1. ICD-10 codes for Charlson comorbidity index.

Diseases	Scores	ICD-10 codes
Myocardial infarction	1	I21.x, I22.x, I25.2, I23.x
Congestive heart failure	1	I09.9, I11.0, I13.0, I13.2, I25.5, I42.0, I50.x
Peripheral vascular disease	1	I73.x, I77.x, I79.x, Z95.8, Z95.9
Cerebrovascular disease	1	G45.x, G46.x, H34.0, I60.x – I69.x
Dementia	1	F00.x–F03.x, F05.1, G30.x, G31.1
Chronic pulmonary disease	1	I27.8, I27.9, J40.x–J47.x, J60.x – J67.x, J68.4, J70.1, J70.3, J99.0, J99.1
Rheumatic disease	1	M05.x – M09.x, M12.x, M30.x – M36.x M45.x, M79.x
Peptic ulcer disease	1	K25.x–K28.x
Mild liver disease	1	B18.x, K70.0 – K70.3, K70.9, K73.x, K76.0, K76.1, K76.2, K76.3, K76.4, K76.8, K76.9, Z94.4
Diabetes without chronic complication	1	E10.0, E10.1, E10.6, E10.8, E10.9, E11.0, E11.1, E11.6, E11.8, E11.9, E12.0, E12.1, E12.6, E12.8, E12.9, E13.0, E13.1, E13.6, E13.8, E13.9, E14.0, E14.1, E14.6, E14.8, E14.9
Diabetes with chronic complication	2	E10.2–E10.5, E10.7, E11.2–E11.5, E11.7, E12.2–E12.5, E12.7, E13.2–E13.5, E13.7, E14.2–E14.5, E14.7
Hemiplegia or paraplegia	2	G04.1, G11.4, G60.3, G80.0, G81.x, G82.x, G83.0–G83.5, G83.8, G83.9
Renal disease	2	I12.0, I13.1, N03.x - N05.x, N17.x - N19.x, N20.x, N26.x, N25.0, Z49.0, Z49.2, Z94.0, Z99.2, I13.2, I13.9, Q61.x Q64.x
Any malignancy including lymphoma and leukemia except for skin malignancy	2	C00.x – C42.x, C45.x-C76.x, C81.x – C97.x, D45.x – D47.x
Moderate to severe liver disease	3	I85.0, I85.9, I86.4, I98.2, K70.4, K71.x, K72.x, K74.x, K76.5, K76.6, K76.7,
Metastatic solid tumor	6	C77.x – C80.x
AIDS/HIV	6	B20.x – B24.x

ICD-10 = International Classification of Diseases, the 10th Revision. AIDS = acquired immune deficiency syndrome, HIV = human immunodeficiency virus.

Table S2. Codes for ICU admissions, treatments, and complications.

Diseases	Codes
ICU admissions *	AJ001, AJ003, AJ006, AJ007, AJ010, AJ011, AJ020, AJ100, AJ200, AJ300, AC612
Mechanical ventilation *	M0850, M0857, M0858, M0860, M5830, M5850, M5857, M5858, M5860, M5890
Continuous renal replacement therapy *	O7031- O7035 and O7051 - O7055
Extracorporeal membrane oxygenation *	O1901- O1907
Myocardial infarction †	I21.x, I22.x, I25.2, I23.x
Myocarditis †	I40.x, I41.x, I51.4
Cerebral infarct †	I67.x, I63.x, I64, I65, I66
Cerebral hemorrhage †	I60.x, I62.x, I61.x, I69.x
Liver failure †	K71.x, K72.x
Heart failure	I09.9, I11.0, I13.0, I13.2, I50.x

AIDS = acquired immune deficiency syndrome, HIV = human immunodeficiency virus.
* The Korean National Health Insurance Service codes.
† The International Classification of Diseases, the 10th Revision (ICD-10).

Table S3. Age, Charlson comorbidity index, treatments, and hospital outcomes.

	2009/2010	2010/2011	2011/2012	2012/2013	2013/2014	2014 2015	2015/2016	2016/2017	2017/2018	2018/2019
Number of hospitalized patients	20,465	9,860	11,400	8,247	25,301	34,986	27,951	34,240	71,944	55,758
Age, years *	43.8 ± 17.5	45.9 ± 16.8	53.8 ± 19.5	51.8 ± 19.2	50.4 ± 18.8	55.7 ± 18.8	49.6 ± 17.9	53.3 ± 20.0	57.2 ± 19.2	48.6 ± 19.4
Male, n (%)	8236 (40.2)	3712 (37.6)	3949 (34.6)	2985 (36.2)	8854 (35.0)	11670 (33.4)	9546 (34.2)	11755 (34.3)	25822 (35.9)	20708 (37.1)
Charlson comorbidity index *	0.18 ± 0.89	0.23 ± 0.97	0.30 ± 1.09	0.29 ± 1.07	0.23 ± 0.94	0.31 ± 1.09	0.28 ± 1.05	0.28 ± 1.06	0.3 ± 1.1	0.25 ± 1.02
ICU Admission, n	712	263	207	128	364	615	496	662	1,427	962
Oseltamivir, n (%)	1,655 (8.1)	3,051 (30.9)	3,517 (30.9)	3,027 (36.7)	8,449 (33.4)	13,294 (38.0)	8,227 (29.4)	11,692 (34.1)	39,613 (55.1)	25,117 (45.0)
Steroids, n	799	419	604	348	957	1,426	924	1,253	3,248	1,950
Length of ICU stay, days †	10.0 (4.5–15.5)	9.0 (3.0–15.0)	11.0 (4.8–17.3)	12.0 (5.5–17.5)	10.0 (4.5–15.5)	11.0 (5.5–16.5)	12.0 (5.0–19.0)	9.0 (3.4–14.6)	10.0 (4.0–16.0)	10.0 (4.5–15.5)
Length of hospital stay, days †	5.0 (3.0-7.0)	5.0 (3.0–7.0)	4.0 (2.0–6.0)	4.0 (2.5–5.5)	4.0 (2.5–5.5)	4.0 (2.5–5.5)	4.0 (2.5–5.5)	4.0 (2.5–5.5)	4.0 (2.5–5.5)	4.0 (2.5–5.5)
ICU deaths, n	92	62	27	15	45	89	92	83	284	179
Hospital deaths, n	135	89	61	30	91	216	169	184	610	314

CRRT = continuous renal replacement therapy, ECMO = extracorporeal membrane oxygenation, ICU = intensive care unit, MV = mechanical ventilation.

* Means ± standard deviations

† Medians (interquartile ranges).

Table S4. Underlying comorbidities of enrolled patients.

Variables	2009/ 2010	2010/ 2011	2011/ 2012	2012/ 2013	2013/ 2014	2014/ 2015	2015/ 2016	2016/ 2017	2017/ 2018	2018/ 2019	Total
Number of patients	20465	9860	11399	8246	25301	34985	27951	34239	71944	55758	300148
Myocardial infarction	34	20	25	25	52	104	62	69	182	128	701
Congestive heart failure	82	40	114	84	157	319	228	314	847	502	2687
Peripheral vascular disease	32	15	37	32	66	138	62	115	252	175	924
Cerebrovascular disease	189	126	202	124	321	638	414	565	1372	783	4734
Dementia	105	39	155	74	213	503	288	476	1203	586	3642
COPD	391	300	534	348	881	1538	1133	1294	2886	1898	11203
Rheumatologic disease	118	107	194	182	463	863	714	754	1510	1119	6024
Peptic ulcer	163	161	243	152	354	673	420	472	920	593	4151
Mild liver disease	244	187	287	196	451	971	797	855	1802	876	6666
Diabetes without complications	305	223	311	282	690	1350	883	1116	2684	1607	9451
Diabetes with complications	144	67	100	70	185	338	227	277	599	325	2332
Hemiplegia or paraplegia	104	51	73	42	87	156	135	203	467	266	1584
Renal disease	125	80	101	79	176	355	246	341	758	530	2791
Malignancy, including leukemia and lymphoma	295	133	152	130	340	541	312	498	1046	728	4175
Moderate or severe liver disease	90	43	54	42	79	186	127	149	323	248	1341
Metastatic solid tumor	62	35	39	27	61	85	75	91	213	142	830
AIDS/HIV	1	1	1	0	1	5	0	2	1	2	14

AIDS = acquired immune deficiency syndrome, COPD = chronic obstructive pulmonary disease, HIV = human immunodeficiency virus.

Table S5. Annual numbers and rates of hospitalization by age groups.

Age groups		2009/ 2010	2010/ 2011	2011/ 2012	2012/ 2013	2013/ 2014	2014/ 2015	2015/ 2016	2016/ 2017	2017/ 2018	2018/ 2019	Total
20s *	n	5,807	1,987	1,246	1,023	3,639	3,038	3,589	4,810	6,881	11,231	43,251
	Incidence †	76.45	26.59	16.93	14.04	49.89	41.42	48.68	64.96	92.84	151.53	58.50
30s	n	3,800	2,168	2,267	1,754	5,325	5,381	6,597	5,669	9,126	10,783	52,870
	Incidence †	45.65	26.35	27.87	21.87	67.86	70.08	87.51	76.73	125.73	151.63	68.20
40s	n	3,048	1,548	1,474	1,266	3,855	5,073	4,778	4,564	8,922	8,517	43,045
	Incidence †	34.97	17.77	16.92	14.46	43.75	57.76	54.82	52.82	105.07	102.17	49.66
50s	n	3,715	2,058	1,878	1,323	4,440	6,693	4,755	5,825	13,746	8,440	52,873
	Incidence †	55.23	28.68	24.92	16.99	55.50	82.13	57.65	69.93	163.10	99.076	67.01
60s	n	1,948	989	1,402	902	2,855	5,098	3,533	4,722	11,755	7,118	40,322
	Incidence †	47.60	23.83	33.24	20.77	63.03	105.67	68.59	86.83	205.44	117.74	83.11
70s	n	1,628	811	1,816	1,212	3,163	5,229	2,791	4,454	10,613	5,117	36,834
	Incidence †	64.18	30.36	63.82	40.47	102.65	167.01	87.94	136.12	312.19	145.97	120.33
80s	n	473	283	1,138	648	1,794	3,816	1,729	3,504	9,117	3,956	26,458
	Incidence †	58.39	32.81	123.75	65.84	168.71	329.16	137.38	258.62	628.71	254.28	231.71
90s	n	46	16	179	119	230	658	179	692	1,784	596	4,499
	Incidence †	47.33	15.08	152.19	90.43	160.43	430.73	109.70	390.93	919.67	279.14	300.68
Total incidence	n	20,465	9,860	11,400	8,247	25,301	34,986	27,951	34,240	71,944	55,758	300,152
	Incidence ‡	52.61	25.04	28.61	20.46	62.06	84.89	67.15	81.51	169.86	130.59	73.37

* Included those aged 19 years.
† Hospitalization rates per 100,000 population.
‡ Age-adjusted hospitalization rates per 100,000 population.

Table S6. Numbers of hospitalizations by regions across South Korea.

Regions	2009/ 2010	2010/ 2011	2011/ 2012	2012/ 2013	2013/ 2014	2014/ 2015	2015/ 2016	2016/ 2017	2017/ 2018	2018/ 2019
Total number	20,465	9,860	11,400	8,247	25,301	34,986	27,951	34,240	71,944	55,758
Busan	2,484	1,240	1,140	918	3,060	4,425	3,149	3,563	6,970	4,893
Chungbuk	694	205	306	360	1,092	1,141	986	1,080	1,932	1,383
Chungnam	805	418	601	395	1,236	1,491	1,142	1,130	3,175	2,129
Daegu	772	567	446	307	1,019	1,459	1,215	1,174	2,490	1,480
Daejeon	633	269	100	95	478	667	743	767	1,606	1,224
Gangwon	728	367	394	170	637	840	600	731	1,441	1,245
Gwangju	861	589	519	577	1,588	2,515	2,024	2,560	4,484	4,095
Gyeongbuk	806	422	599	516	962	1,627	1,160	1,177	3,513	2,384
Gyeongi	3,569	1,429	1,736	914	3,545	4,310	3,471	4,081	10,288	9,260
Gyeongnam	2,457	1,347	2,298	1,738	4,236	5,615	4,410	5,577	11,108	6,998
Incheon	630	247	379	231	1,006	1,100	1,427	1,388	3,115	2,532
Jeju	172	119	106	98	343	511	394	438	1,110	619
Jeonbuk	989	464	462	319	1,203	1,990	1,176	2,630	4,831	4,323
Jeonnam	1,250	634	972	639	1,694	3,305	2,058	3,193	7,325	6,392
Seoul	2,754	1,032	1,012	671	2,407	2,883	2,898	3,319	5,782	5,064
Ulsan	861	511	330	299	763	1,066	1,047	1,299	2,696	1,717
Sejong	NA	NA	NA	NA	32	41	51	133	78	20

Table S7. Critical care resource utilization.

Variables	N/Rate	2009/ 2010	2010/ 2011	2011/ 2012	2012/ 2013	2013/ 2014	2014/ 2015	2015/ 2016	2016/ 2017	2017/ 2018	2018/ 2019	p value
ICU Admission	n	712	263	207	128	364	615	496	662	1,427	962	< 0.001
	rate *	1.83	0.67	0.52	0.32	0.89	1.49	1.19	1.58	3.37	2.25	
Steroid	n	799	419	604	348	957	1,426	924	1,253	3,248	1,950	< 0.001
	rate *	2.05	1.06	1.52	0.86	2.35	3.46	2.22	2.98	7.67	4.57	
Mechanical ventilation	n	246	156	78	54	108	200	211	199	524	353	< 0.001
	rate *	0.63	0.40	0.20	0.13	0.26	0.49	0.51	0.47	1.24	0.83	
CRRT	n	40	25	9	5	16	26	42	32	95	88	< 0.001
	rate *	0.10	0.06	0.02	0.01	0.04	0.06	0.10	0.08	0.22	0.21	
ECMO	n	8	3	2	0	2	6	26	6	9	16	< 0.001
	rate *	0.02	0.01	0.01	0.00	0.00	0.01	0.06	0.01	0.02	0.04	
Vasopressors	n	137	57	65	26	56	79	155	375	962	589	< 0.001
	rate *	0.35	0.14	0.16	0.06	0.14	0.19	0.37	0.89	2.27	1.38	

CRRT = continuous renal replacement therapy, ECMO = extracorporeal membrane oxygenation, ICU = intensive care unit.
* Rates per 100,000 population, compared by Cochran-Armitage trend test.

Table S8. Annual numbers and rates of in-hospital deaths.

Age groups		2009/ 2010	2010/ 2011	2011/ 2012	2012/ 2013	2013/ 2014	2014/ 2015	2015/ 2016	2016/ 2017	2017/ 2018	2018/ 2019	Total
20s *	n	6	1	0	0	0	2	1	0	2	2	14
	Incidence †	0.08	0.01	0.00	0.00	0.00	0.03	0.01	0.00	0.03	0.03	0.02
30s	N	9	4	1	0	1	0	5	0	0	5	25
	Incidence †	0.11	0.05	0.01	0.00	0.01	0.00	0.07	0.00	0.00	0.07	0.03
40s	N	14	6	5	1	0	3	4	4	0	7	44
	Incidence †	0.16	0.07	0.06	0.01	0.00	0.03	0.05	0.05	0.00	0.08	0.05
50s	N	20	16	3	5	7	8	16	5	20	24	124
	Incidence †	0.30	0.22	0.04	0.06	0.09	0.10	0.19	0.06	0.24	0.28	0.16
60s	N	26	21	4	2	14	18	25	26	38	32	206
	Incidence †	0.64	0.51	0.09	0.05	0.31	0.37	0.49	0.48	0.66	0.53	0.42
70s	N	40	21	13	6	33	50	49	55	149	82	498
	Incidence †	1.58	0.79	0.46	0.20	1.07	1.60	1.54	1.68	4.38	2.34	1.63
80s	N	15	19	26	11	30	92	59	62	269	121	704
	Incidence †	1.85	2.20	2.83	1.12	2.82	7.94	4.69	4.58	18.55	7.78	6.17
90s	N	5	1	9	5	6	43	10	32	132	41	284
	Incidence †	5.14	0.94	7.65	3.80	4.19	28.15	6.13	18.08	68.05	19.20	18.98
Total incidence	N	135	89	61	30	91	216	169	184	610	314	1,899
	Incidence ‡	0.35	0.23	0.15	0.07	0.22	0.52	0.41	0.44	1.44	0.74	0.46

* Included those aged 19 years.

† In-hospital deaths rates per 100,000 population,

‡ Age-adjusted hospital death rates per 100,000 population

Table S9. Annual incidence of ILI visits per 1,000 patients and influenza virus isolates by subtypes*.

	**2009**	**2010**	**2011**	**2012**	**2013**	**2014**	**2015**	**2016**	**2017**	**2018**
ILI visits per 1,000 patients †	13.15	23.89	3.7	3.3	15.3	8.9	9.0	86.2	71.8	73.3
Number of isolated subtypes										
A(H1N1)	1,471	0	0	0	0	0	0	0	0	0
A(H1N1)pdm09	3,979	1,708	727	21	322	343	174	577	44	575
A(H3N2)	1,668	112	419	1,742	1,273	641	836	520	635	660
A(not subtyped)	-	-	-	-	-	-	1	0	0	0
B	73	1,704	65	1,787	178	1,027	603	667	625	799
Total number	7,191	3,524	1,211	3,550	1,773	2,011	1,614	1,764	1,304	2,034

ILI = influenza-like illness, A(H1N1)pdm09 = 2009 A(H1N1) pandemic influenza.

* Data were extracted from Infectious Diseases Surveillance Yearbook by the Korea Disease Control and Prevention Agency (available at http://www.kdca.go.kr/npt/biz/npp/portal/nppPblctDtaView.do?pblctDtaSeAt=1&pblctDtaSn=2139).

† Korea influenza surveillance program using influenza-like illness.

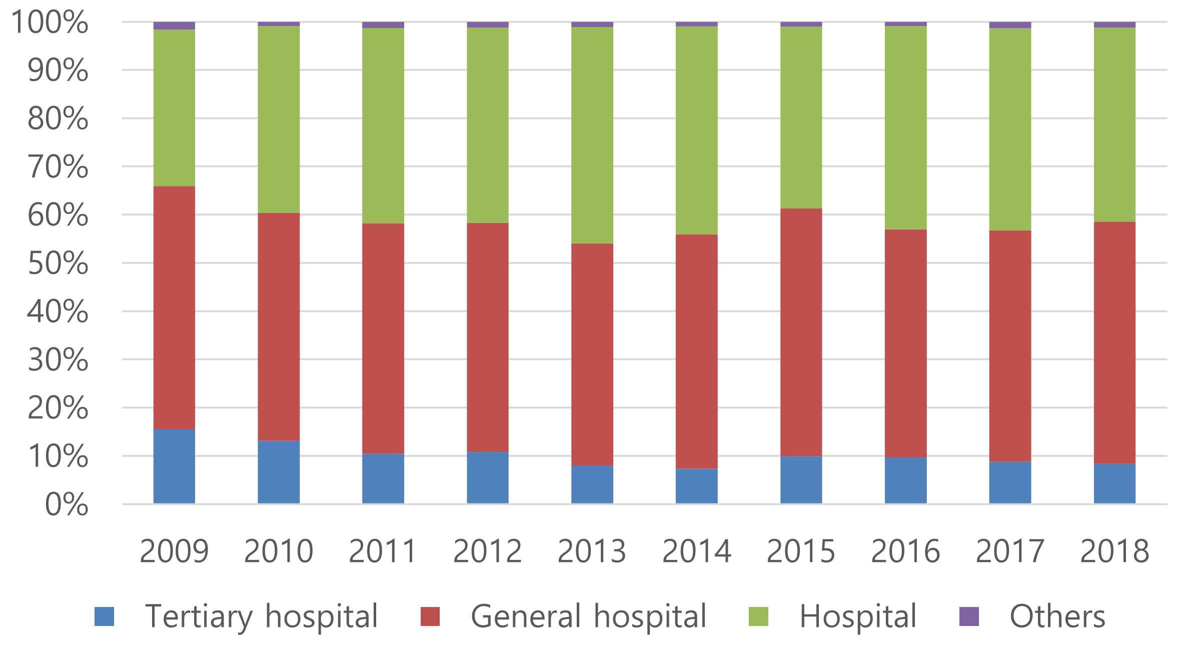

Figure S1. Distribution of hospital types

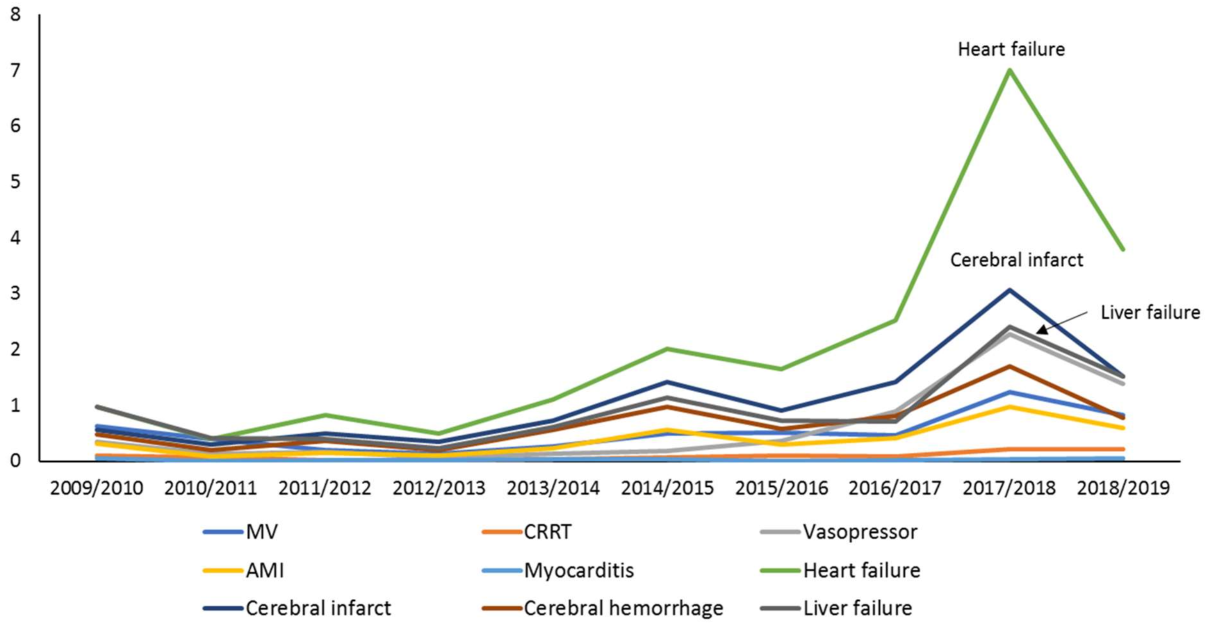

Figure S2. Incidence of complications per 100,000 population. AMI = acute myocardial infarct, MV = mechanical ventilation, CRRT = continuous renal replacement therapy
